# Supplementary material for: Tissue-resident memory T cells populate the human brain
Source: Nat Commun. 2018 Nov 2;9:4593. doi: 10.1038/s41467-018-07053-9 (PMC6214977; doi:10.1038/s41467-018-07053-9)
Supplement: Supplementary file 1 — Supplementary Files [file 41467_2018_7053_MOESM1_ESM.pdf]

Supplementary Information File

Tissue-resident memory T cells populate the human brain

Smolders et al.

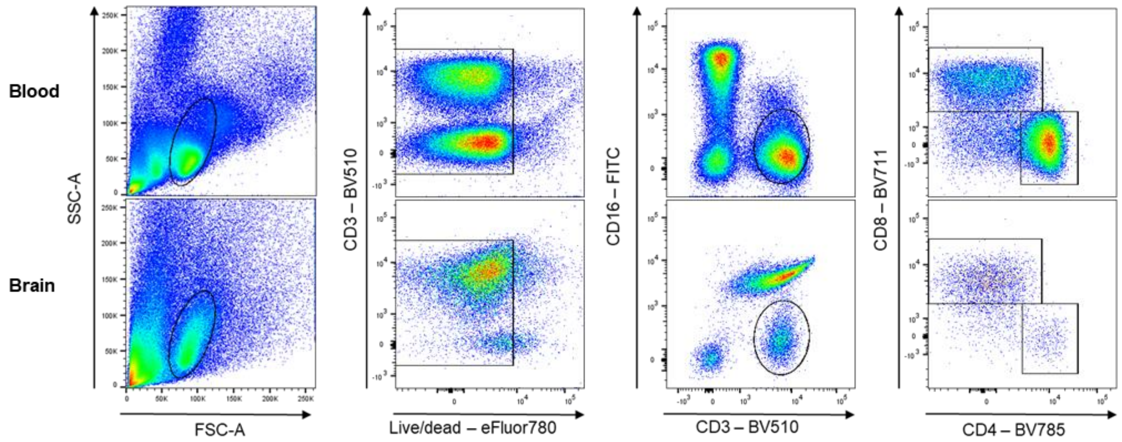

Supplementary Figure 1. Example of CD8⁺ and CD4⁺ T-lymphocyte gating strategy. Paired blood- and brain-derived cells of a donor with Parkinson's disease. Panels from left to right show subsequent within-gate events. Exclusion of duplets (SSC-H*SSC-W and FSC-H*FSC-W) was performed but is not shown. Note the abundant viable brain cell population in the $CD3^+$ fluorescence range, which were not T cells and were gated from the T-cell population by either including an empty channel or by including non-T-cell markers or supra-physiological expression of T-cell markers in the gating procedure (in this example, CD16). Cells and markers were back-gated to ensure no relevant cell populations were lost by this strategy.

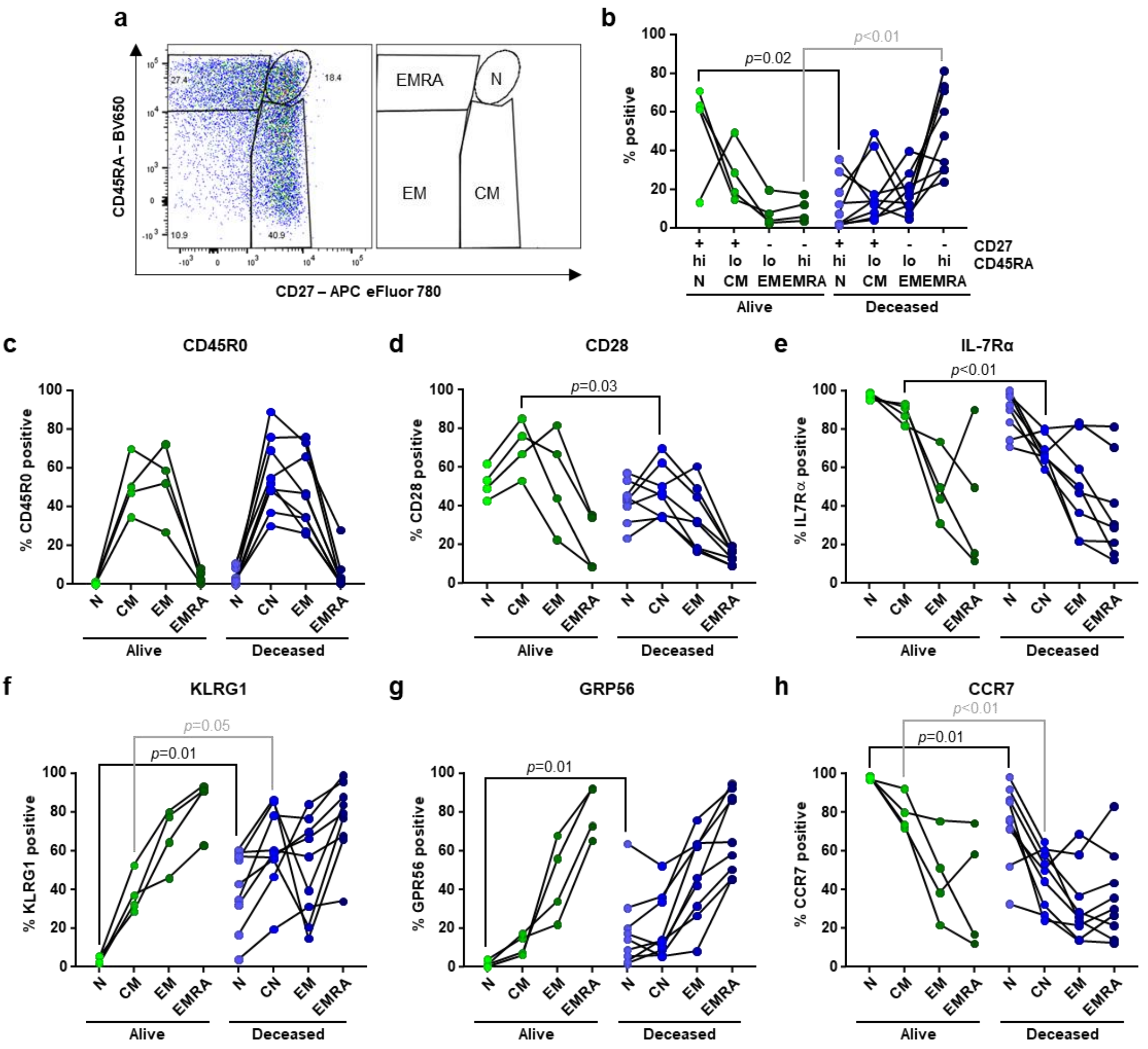

Supplementary Figure 2. T cells from deceased donors shows a further differentiated phenotype compared to healthy controls, with a similar profile of differentiation markers. Comparison of PBMC from n=4 healthy controls with post-mortem blood of n=8–9 brain donors. **(a)** Gating strategy. **(b)** Quantification of $CD8^+$ T cells with a $CD27^+CD45RA^+$ (N, naïve), $CD27^+CD45RA^-$ (CM, central memory), $CD27^-CD45RA^-$ (EM, effector memory), and $CD27^-CD45RA^+$ (EMRA, $CD45RA^+$ effector memory) phenotype[1]. **(c–h)** Expression of CD45R0, CD28, IL-7Rα, KLRG1, GPR56, and CCR7 stratified for CD27 and CD45RA co-expression, respectively. *p*-values show Mann–Whitney U test; the absence of brackets indicates the absence of a significant difference. Note the general similar profiles, although some differences exist. It is not clear whether these are due to post-mortem effects, age, (terminal) disease state, or the general higher level of antigen experience in brain donors.

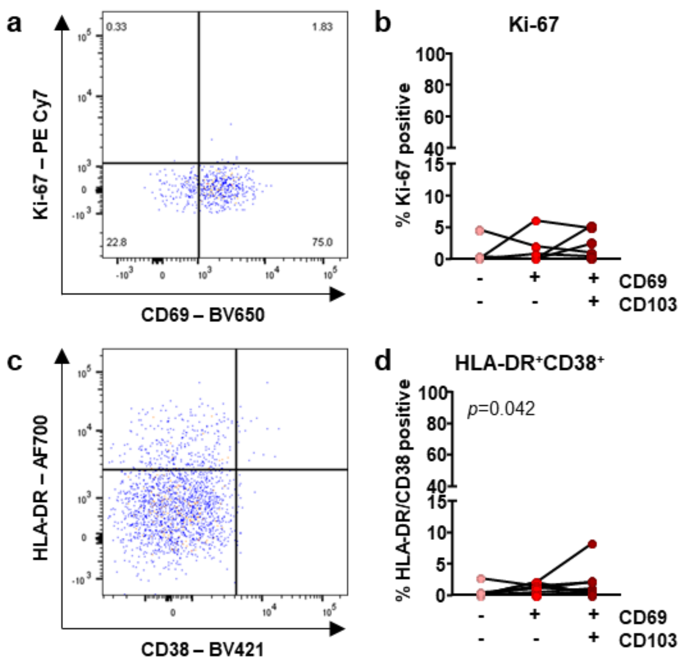

Supplementary Figure 3. Brain CD8$^+$ T cells contain very low proportions of Ki-67$^+$ and HLA-DR$^+$CD38$^+$ cells. Staining of brain CD8$^+$ T cells for Ki-67 **(a)** and HLA-DR/CD38 co-expression **(c)** in a donor, with quantification (**(b)** and **(d)**, respectively). *p*-values show Friedman test (post-hoc Wilcoxon signed ranks showed no significant differences); no brackets indicate no significant difference. Note the substantial CD69 expression, while few cells are either Ki-67$^+$ or HLA-DR$^+$CD38$^+$.

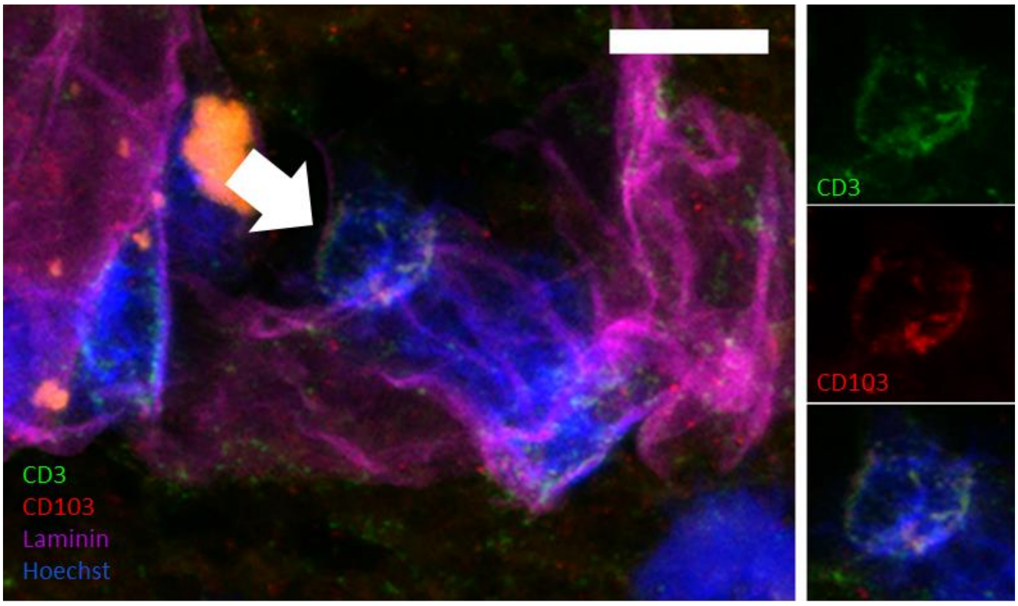

Supplementary Figure 4. CD3 CD103 double-positive T cells reside in the perivascular space. Triple immunofluorescent labeling for CD3 (green), CD103 (red), and laminin (magenta) with Hoechst. The arrow marks a CD3 and CD103-positive cell in close association with the perivascular space (scale bar = 10 µm).

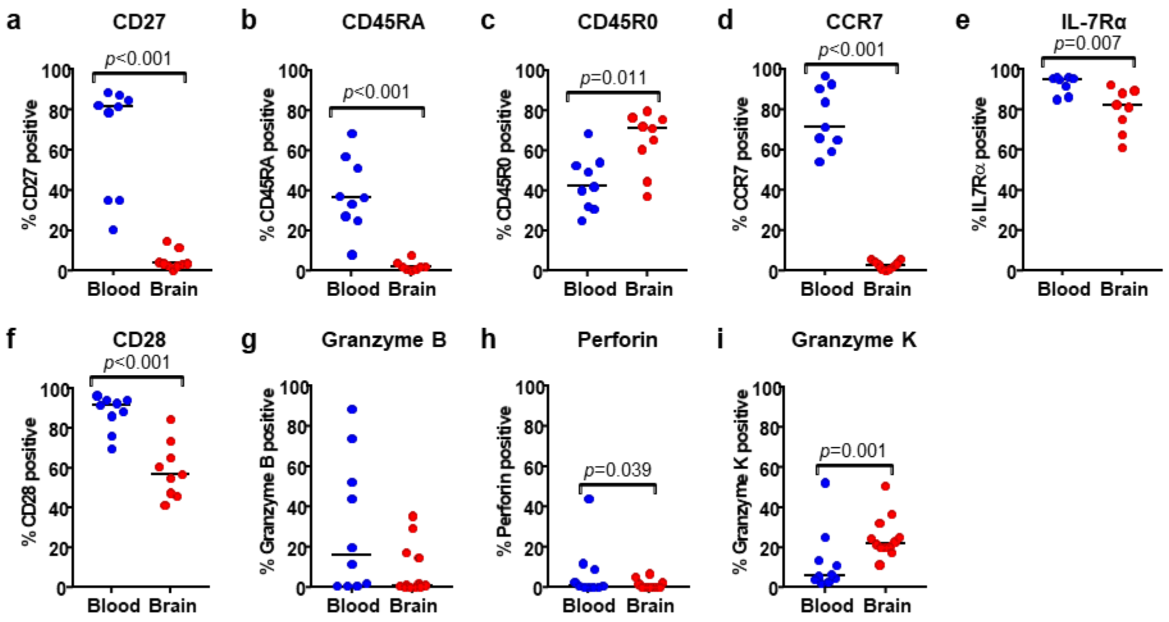

Supplementary Figure 5. Differentiated CD4⁺ T cells populate the human brain. Quantification of CD27, CD45RA, CD45R0, CCR7, IL-7Rα, CD28, granzyme B, perforin, and granzyme K expression by brain-derived CD4⁺ T cells and peripheral blood-derived CD4⁺ T cells. *p*-values show Mann–Whitney U test, the absence of brackets indicates no significant difference.

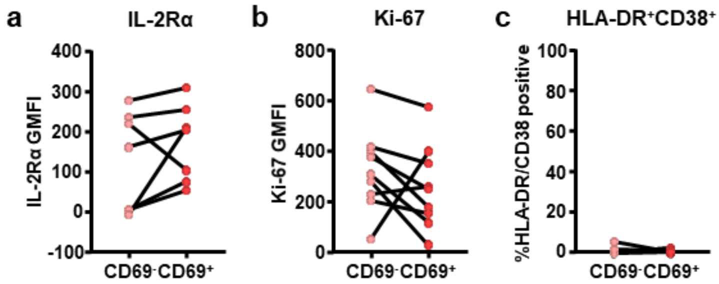

Supplementary Figure 6. Human brain CD4⁺ T-cell expression of activation markers IL-2Rα, Ki-67, and HLA-DR/CD38, stratified for CD69 expression. The absence of brackets indicates the absence of a significant difference tested with the Wilcoxon signed ranks test.

Supplementary Table 1. Specification of antibodies used for flow cytometry

Specificity	Clone	Fluorochrome	Manufacturer
CD3	UCHT1	V500	BD
CD3	7D6	PE Cy5.5	eBioscience
CD4	SK3	BV421	BD
CD4	OKT4	BV510	Biolegend
CD4	SK3	APC	BD
CD8	RPA-T8	BV711	Biolegend
CD8	RPA-T8	BV785	Biolegend
CD16	CLB-Fc-gran/1, 5D2	FITC	Sanquin
CD25	BC96	APC-eFluor780	eBioscience
CD27	O323	APC-eFluor780	eBioscience
CD38	HIT2	BV421	Biolegend
CD45RA	HI100	BV650	BD
CD45R0	UCHL1	BV785	Biolegend
CD49a/ITGA1	TS2/7	APC-Vio770	Miltenyi Biotec
CD49D/VLA4	9F10	PE-Cy7	Biolegend
CD57	HCD57	PE	Biolegend
CD69	FN50	BV650	Biolegend
CD69	FN50	BV421	Biolegend
CD69	FN50	BV786	BD
CD103	Ber-Act8	BUV395	BD
CD103	Ber-Act8	PerCP-eFluor710	eBioscience
IL-7Rα (CD127)	eBioRDR5	PE-Cy7	eBioscience
CCR5 (CD195)	Hek/1/85a	AF700	Biolegend
CCR7 (CD197)	150503	BUV395	BD
CXCR3 (CD183)	G025H7	PerCP-Cy5.5	Biolegend
CXCR6 (CD186)	K041E5	PE-Cy7	Biolegend
CX_3CR1	2A9-1	PE	eBioscience
Eomes	WD1928	AF647	eBioscience
GM-CSF	BVD2-21C11	PE	BD
GPR56	CG4	PE	H.H. Lin, Chang Gung University, Tao-Yuan, Taiwan[2]
Granzyme B	GB11	AF700	BD
HLA-DR	LN3	AF700	eBioscience
Hobit[A]	Hobit/1	PE	K.P.J.M. van Gisbergen, Sanquin Research, Amsterdam, Netherlands[3]
IFN-γ	25723.11	FITC	BD
IL-17A	BL-168	BV421	Biolegend
Ki-67	B56	PE-Cy7	BD
KLRG1	13A2	AF488	H. Pircher, University of Freiburg, Germany[4]
Perforin	DG9	PerCP-eFluor710	eBioscience
T-bet	4B10	BV421	Biolegend
TNF-α	Mab11	PE-cy7	eBioscience
[A]Mouse IgM antibody detected with mouse-anti-mouse IgM-PE			

Supplementary References

1. Hamann, D. *et al.* Phenotypic and functional separation of memory and effector human CD8+ T cells. *J. Exp. Med.* **186,** 1407–18 (1997).
2. Peng, Y.-M. *et al.* Specific expression of GPR56 by human cytotoxic lymphocytes. *J. Leukoc. Biol.* **90,** 735–740 (2011).
3. Vieira Braga, F. A. *et al.* Blimp-1 homolog Hobit identifies effector-type lymphocytes in humans. *Eur. J. Immunol.* **45,** 2945–58 (2015).
4. Marcolino, I. *et al.* Frequent expression of the natural killer cell receptor KLRG1 in human cord blood T cells: correlation with replicative history. *Eur. J. Immunol.* **34,** 2672–80 (2004).
